# A randomized clinical trial comparing 3 different replacement regimens of vitamin D in clinically asymptomatic pediatrics and adolescents with vitamin D insufficiency

**DOI:** 10.1186/s13052-016-0314-z

**Published:** 2016-12-07

**Authors:** Iman M. Talaat, Naglaa M. Kamal, Hamed A. Alghamdi, Abdulla A. Alharthi, Mohamed A. Alshahrani

**Affiliations:** 1 Pediatric department, Faculty of Medicine, Ain Shams University, Cairo, Egypt; 2 Pediatric department, Faculty of Medicine, Cairo University, Cairo, Egypt; 3 Pediatric department, Alhada Armed Forces Hospital, Taif, Saudi Arabia; 4 Director of Alhada Armed Forces Hospital, Taif, Saudi Arabia; 5 College of Medicine, Taif University, Taif, Saudi Arabia; 6 Laboratory department, Prince Mansour Military Community Hospital, Taif, KSA Saudi Arabia

**Keywords:** Vitamin D insufficiency, Vitamin D deficiency, 25 (OH) D, Replacment regimens

## Abstract

**Background:**

Pediatric and Adolescent populations both have special needs for vitamin D especially for growing bone. Inadequate vitamin D is defined as 25 (OH) D(25hydroxy vitamin D) < 30 ng/ml.

**Methods:**

We conducted a randomized, controlled clinical trial from July 2014 over 1 year, aiming to assess the changes in 25 (OH) D and biochemical outcome on calcium and PTH(parathyroid hormone) using 3 different regimens of vitamin D replacement. Initial and 4 month 25 (OH) D, calcium, PTH and 12 month 25 (OH) D levels were assayed. Participants divided into 3 groups: 1) given 400 IU daily, 2) given 45000 IU weekly for 2 months then 400 IU daily, 3) given 2000 IU daily for 3 months then 1000 IU daily.

**Results:**

The results showed significant difference between the 3 groups as regards 25 (OH) D at 4 and 12 months (*P* < 0.001). Regimens used in group 2 and 3 caused increase in 25 (OH) D after 4 month (median increase is 225% and 200% respectively). 25 (OH) D dropped in group 1 and 2 (median decrease is 42 and 53% respectively) but continued to increase in group 3 (median change is 6%). In group 2 serum calcium median change was 1.2% with few cases of hypercalcuria. 94.9, 76.1 and 7.7 are the percent of vitamin D deficient participants in groups 1, 2 and 3 respectively after 12 months follow up.

**Conclusion:**

We advise as a replacement for vitamin D insufficiency, low loading dose with high maintaince dose rather than the opposite to achieve steady increase in serum 25 (OH) D with no hypercalcemic side effects.

## Background

Vitamin D is obtained either by ingestion or cutaneous production. When skin is exposed to ultraviolet B radiation, 7-dehydrocholesterol is converted to vitamin D3 (cholecalciferol). Dietary sources may provide either vitamin D3 or vitamin D2 (ergocalciferol) [1]. Combining low intake with indoor lifestyle and sun-avoiding behaviors, it is not surprising that low vitamin D status is endemic [2, 3]. Cohort studies from Saudi Arabia showed high prevalence of vitamin D deficiency ranging from 21.9 to 81% among children and adolescent school girls respectively [4]. Given the high rate of bone development early in life, adequate serum concentrations of vitamin D are crucial for the developing child. There has also been a piquing interest in vitamin D in pediatric patients due to the recent epidemiologic reports suggesting that vitamin D may protect against autoimmune disease and play a role in innate immunity [5]. A serum calcidiol 25-(OH)D concentration is a widely used marker of vitamin D status, because it is considered to be a reasonable reflection of total body exposure to vitamin D. Circulating 25(OH)D represents both endogenous vitamin D synthesis in the skin and dietary intake of vitamin D [6]. The serum concentration that constitutes vitamin D deficiency or insufficiency is controversial and not well supported by clinical trials, especially in the pediatric population [7]. The American Academy of Pediatrics(AAP) and the Institute of Medicine (IOM) both define vitamin D insufficiency as 25(OH)D concentrations < 20 ng/mL in the pediatric population [6, 8]. In contrast, the Endocrine Society and the National Kidney Foundation Kidney Disease Outcomes Quality Initiative (KDOQI) guidelines both classify insufficiency as 25(OH) D concentrations < 30 ng/mL. The Endocrine Society defines deficiency as < 20 ng/mL and KDOQI defines deficiency as < 15 ng/mL [9, 10]. As long as reliance on sunlight exposure to produce vitamin D in the skin is not recommended [11] and the natural food sources of vitamin D are not particularly kid-friendly [12], this means that food fortification or vitamin D supplementation are the alternative solutions. As the meta-analysis of 167 studies done by Cranney et al. [13] showed that the largest body of evidence on vitamin D treatment strategies and bone health was in older adults with a lack of studies in premenopausal women, infants, children and adolescents. Guidelines for the treatment of vitamin D insufficiency in healthy children included the use of a wide range of cumulative vitamin D doses (84,000 to 600,000 IU) and recommend the higher doses for adolescents [6]. We conducted this study aiming to assess different physician approaches for vitamin D insufficiency in children and adolescents. Reviewing literature showed controversy about need for vitamin D screening and replacement if clinically asymptomatic and there was no standard regimen existing for repletion in such age group and no studies to assess capability of different regimens to maintain vitamin D sufficiency over a longer period.

## Methods

### Study design and participants

This is a randomized, controlled clinical trial on children and adolescents 2–18 years of age recruited from 3 pediatric clinics in different centers of Armed forces hospitals-Taif region-KSA (Latitude 21.43°N),during routinely scheduled well-child visits from July 2014 over I year. We excluded infants less than 2 years old due to lack of BMI Z score for this age and their large consumption of milk and milk formulas compared to other age groups. The study started simultaneously in the 3 centers to exclude the effect of seasonal variation on the initial assay of vitamin D. Race, ethnicity, income, physical activity, sun exposure and outdoor activity are variables that potentially affect vitamin D level were grossly uniform in all included children and adolescents. We excluded who had chronic illness, failure to thrive or severe developmental delay, darkly pigmented children and adolescents, those with symptoms can be attributed to vitamin D deficiency and who received multivitamins or vitamin D supplements in the previous 6 months. The research assistants also obtained physical measurements of the children and adolescents as weight and height using SECA Model 767 scale. BMI Z score calculation was done based on the center for Disease control (CDC) charts.

Venous blood sampling was performed by trained phlebotomists for all participants initial, after 4 months and 1 year of initiation of the study. Urine samples were taken for all participants after 4 months of initiation of study.

Samples were daily sent to the Clinical Biochemistry Laboratory at Al-Hada Armed Forces Hospital.

### Sample collection

- Venous blood was collected into K2-EDTA, 3.2% (w/v) citrate and serum tubes (BD) through vena punction using 21 gauge. Sera were obtained by centrifugation and were kept at −80 °C until usage.
- Estimation of calcium metabolism (blood and urine)
- Serum levels of calcium were measured using an ARCHITECT/AEROSET System (Abbott Diagnostics Division, Abbott Laboratories).
- Serum intact Parathyroid hormone (PTH) was estimated in a two-steps sandwich immunoassay on ARCHITECT c Systems (Abbott Diagnostics) using chemiluminescent microparticle immunoassay (CMIA) technology.
- Measurement of 25-hydroxyvitamin D

Serum 25(OH)D was quantified by direct competitive chemiluminescence immunoassay (CLIA) using the LIAISON 25 OH Vitamin D TOTAL Assay and DiaSorin LIAISON automated analyzer (DiaSorinInc, Stillwater, MN, USA). The assay was performed according to the manufacturer’s instructions. Briefly, the assay uses magnetic particles (solid phase) coated with antibody against 25(OH) D and 25(OH)D conjugated to an isoluminol derivative (tracer). During the first incubation phase (10 min), 25(OH) D was dissociated from binding protein by buffer containing 10% ethanol and then binds to the anti-25(OH) D antibody on the magnetic particles. After 10 min incubation, the conjugated 25(OH) D was added and allowed to incubate for another 10 min again. The magnetic particles were then washed of all excess liquid and unbound material. A starter reagent was added to initiate a flash chemiluminescent reaction of the conjugated isoluminol derivative, which was then measured by a photomultiplier. The amount of light measured was inversely proportional to the concentration of 25(OH) D present in the sample. A two point calibration curve, consisting of serum containing known 25(OH) D concentrations, was constructed. Unknowns were obtained by comparison with the calibration curve.

### Protocol of the study

In our study, inadequate vitamin D is defined as vitamin D <30 ng/ml. Vitamin D deficiency, insufficiency and sufficiency were defined as a serum 25 (OH) D level of <20 ng/ml, 21 to 29 ng/ml, and ≥30 ng/ml, respectively based on the Endocrine Society reference [9, 10]. Vitamin D toxicity defined as 25 (OH) D >150 ng/ml based on American Academy of Pediatrics 2008 (AAP) and IOM [14].

Both vitamin D2 and vitamin D3 increase serum 25(OH) D concentrations. In adults, some [15, 16] but not all [17] studies have suggested that vitamin D3 is more effective in increasing serum 25(OH) D concentrations than in vitamin D2, despite similar absorption in healthy subjects [17]. This difference between ergocalciferol and cholecalciferol may relate to variations in binding to vitamin D-binding protein [18] and more rapid clearance of vitamin D2 [13]. So, in our study, we preferred to use vitamin D 3 supplements. Repeat evaluation of 25(OH) D at earlier time points seems inappropriate as it takes 3–6 months for serum 25(OH) D to plateau following initiation of supplementation [19] that is why our initial evaluation was 4 months after initiation of the study.

Because different treatment modalities are reported in literature that vary according to dose and duration [10, 20], in this study we aimed to compare between 3 different ways of treating vitamin D insufficiency, first using only replacement with the recommended dietary allowance (RDA) (group 1) because participants are asymptomatic, second is using high loading dose of vitamin D3 and small maintaince dose as used in (group 2) and third one using low loading dose of vitamin D3and high maintaince dose as used in (group 3).

Six-hundreds-thirty-seven children and adolescents were included and divided into 3 groups: Group (1): including 196 participants with 400 IU daily vitamin D3 supplementation given. Group (2): including 247 participants with inadequate vitamin D given vitamin D3 supplementation 45,000 IU vitamin D3 oral weekly over 2 months period followed by maintaince oral dose 400 IU almost daily. Group (3): including 194 participants with inadequate vitamin D given vitamin D3 supplementation 2000 IU oral daily over 3 months followed by maintaince oral dose 1000 IU for all participants almost daily. To guarantee the compliance to vitamin D intake in the 3 groups, we included only participants who had regular vitamin D refill confirmed by the pharmacy electronic system.

### Statistical analysis

Data were statistically described in terms of mean ± standard deviation (±SD), median and range, or frequencies (number of cases) and percentages when appropriate. No variables have missing data. Comparison of numerical variables between the study groups was done using Student *t* test for independent samples. For comparing categorical data, Chi square (*χ*2) test was performed.

Exact test was used instead when the expected frequency is less than 5. *p* values less than 0.05 was considered statistically significant. All statistical calculations were done using computer program SPSS (Statistical Package for the Social Science; SPSS Inc., Chicago, IL, USA) release 15 for Microsoft Windows (2006). Being the primary outcomes, power analysis was based on the comparison of vitamin D, calcium, PTH and Ca/creatinine ratio between the three study groups. The mean of each parameter in each group was entered and the highest SD was selected as intragroup variation parameter. The effect size was calculated then entered to calculate the power of our statistical results. Omnibus one way analysis of variance test was used in the analysis with type I error probability equals 0.05. Calculations were done using G*Power software version 3.1.2 for MS Windows, Franz Faul, Kiel University, Germany.

## Results

The study included 637 participants, 314 males (49.3%) and 323 females (50.7%). The mean age is 8.51 ± 3.527. Base line characteristics of the participants in each group are shown in Table 1. Power calculation supported the validity of statistical estimation of 3 timepoints of vitamin D on the population. Initial 25 (OH) D was < 30 ng/dl in all participants with a mean 16.13 ± 7.865 with significantly higher initial 25 (OH) D in group 1 compared to groups 2 and 3 (*P* < 0.01) (Table 1), and parallel changes in initial PTH in group 1 compared to groups 2 and 3. 81.6% showed initial 25 (OH) D deficiency in group 1 compared to 99.6 and 100% in groups 2 and 3 respectively (Table 3). No noted significant difference between 3 groups and in between individual groups as regards initial calcium, BMI Z score (Table 1).

**Table 1:** Baseline characteristics of participants, comparison between 3 groups as regards age, BMI Z Score, initial {25 (OH)D, calcium, parathyroid hormone}, 4 months{25 (OH)D, calcium, parathyroid hormone, calcium/creatinine ratio} and 12 month 25 (OH)D

	Group 1 (*n*=196)	Group 2 (*n*=247)	Group 3 (*n*=194)	*P* value
Number of females	97 (49.5%)	146 (59.1%)	80 (41.2%)	–
Number of males	99 (50.5%)	101 (40.9%)	114 (58.8%)	–
Age (mean±SD)	8.22±3.47	8.91±3.76	8.21±3.43	0.056
BMI Z score (mean±SD)	0.37±0.88	0.41±0.89	0.23±0.96	0.116
Initial Vitamin D (mean±SD)	21.67±9.53	14.29±5.43	12.88±5.33	<0.001
Initial calcium (mean±SD)	2.45±0.11	2.432±0.12	2.454±0.12	0.089
Initial PTH (mean±SD)	43.99±26.72	53.15±35.62	45.79±28.33	0.004
Vitamin D after Months (mean±SD)	16.94±9.4	47.7±21.37	36.24±7.02	<0.001
Calcium after 4 Months (mean±SD)	2.36±0.1	2.46±0.09	2.36±0.1	<0.001
PTH after 4 Months (mean±SD)	76.81±37.53	36.18±16.6	74.49±32.42	<0.001
Calcium/creatinine ratio (mean±SD)	0.12±0.06	0.18±0.15	0.12±0.06	<0.001
Vitamin D after 12 Months (mean±SD)	10.24±8.26	23.07±12.7	37.7±5.29	<0.001

Normal ranges of Vitamin D [25(OH) D] =30–100 ng/ml, Calcium (Ca) =2.2–2.7 mmol/L, Parathyroid hormone (PTH) =15–68.3 pg/ml, urine calcium/creatinine ratio<0.4 [21]. *P* value considered significant if < 0.05

### Efficacy of vitamin D repletion regimens

There was a significant difference in between 3 groups and individual groups as regards 25 (OH) D (*P* < 0.001) (Table 1). Repletion regimens used in groups 2 and 3 caused significant increase in 25 (OH) D after 4 months in both groups with median increase of 225 and 200% in groups 2 and 3 respectively (Table 2). Concerning 12 months follow up, 25 (OH) D level showed significant difference between 3 groups and each group (*P* < 0.001) (Table 1). Repletion regimens used in group 1 and 2 showed continued drop of 25 (OH) D with median change of 42 and 53% in groups 1 and 2 respectively. But in group 3, 25 (OH) D continued to increase (% change almost 6) (Table 3).

**Table 2:** Comparison between 3 groups as regards percent change in mean vitamin D and serum calcium

Item	Group 1	Group 2	Group 3	*P* value
Initial Vitamin D to 4 month Vitamin D% change (Median and range)	˗23.97 (−63.6 _190.9)	225 (−14.3_1100)	200 (37.5 _880)	<0.001
Vitamin D 4 month to 12 month Vitamin D% change (Median and range)	˗42.31 (−77.8 _ 32.4)	˗53.13 (−83 _225)	5.97 (−36.8 _90.9)	<0.001
Initial Calcium to 4 month Calcium% change (Median and range)	˗3.97 (−14.8 _11)	1.23 (−14.4 _21.8)	˗4.21 (−23.5 _13.6)	<0.001

*P* value considered significant if < 0.05

**Table 3:** Number and percent of vitamin D deficiency, insufficiency, sufficiency and toxicity in the 3 groups

Item	Group 1	Group 2	Group 3	*P* value
Initial Vitamin D				
Deficient (number &%)	160 (81.6%)	246(99.6%)	194 (100%)	<0.001
Insufficient (number &%)	36(18.4%)	1(0.4%)	0 (0%)	
Sufficient (number &%)	0 (0%)	0 (0%)	0 (0%)	
Vitamin D level after 4 Months				
Deficient (number &%)	172(87.8%)	57 (23.1%)	44 (22.7%)	<0.001
Insufficient (number &%)	24 (12.2%)	97(39.3%)	147 (75.8%)	
Sufficient (number &%)	0 (0%)	91(36.8%)	3 (1.5%)	
Toxic (number &%)	0 (0%)	2(0.8%)	0 (0%)	
Vitamin D level after 12 Months				
Deficient (number &%)	186(94.9%)	188 (76.1%)	15 (7.7%)	<0.001
Insufficient (number &%)	10 (5.1%)	47 (19%)	177 (91.2%)	
Sufficient (number &%)	0 (0%)	12 (4.9%)	2 (1%)	
Toxic (number &%)	0 (0%)	0 (0%)	0 (0%)	

*P* value considered significant if < 0.05

After 4 months repletion regimens, 25 (OH) D levels ranged from 4 to 150 ng/ml. The regimens used in groups 2 and 3 corrected vitamin D deficiency by 76.5 and 77.3% respectively (almost equal) with 2 reported cases of vitamin D toxicity in group 2, but 12 month repletion regimen used in group 2 was unable to maintain this rise in vitamin D because again percent of vitamin D deficiency increased by 53% compared to 15% further decrease of vitamin D deficient participants in group 3 (Table 3).

Also, noted the continued drop in 25 (OH) D in group 1 by almost 24% after 4 months and 42% after 12 months (Table 2) and vitamin D deficiency increased to 95% with 12 months follow up and none of participants in group 1 had sufficient vitamin D levels (Table 3).

### Biochemical changes after 4 months repletion

After 4 month treatment serum calcium ranged from 2.12 to 2.7 (reference range 2.2-2.7 mmol/L). There was a significant difference in between the 3 groups, group 1 and 2, group 2 and 3 (*P* < 0.001) with no significant difference in between groups 1 and 3(*p* > 0.05) (Table 1). Serum Calcium after 4 months vitamin D repletion showed continued drop of serum calcium in both groups 1 and 3 with median % change almost the same in both groups (Table 2), which can be explained by the concomitant drop of 25 (OH) D in group 1, however the decrease of calcium in group 3 despite the increase in 25 (OH) D cannot be explained. Calcium/creatinine ratio and PTH after 4 month, both were directly related to 4 months serum calcium level if compared in between the 3 groups and each group individually (Table 1). In group 2, serum calcium showed median increase by 1.2% with few cases of significant hypercalcuria (up to 1.5%) but still no reported hypercalcaemia in the participants (highest level reported is 2.7 mmol/L).

### Comparison between male and female participants

Our study showed that initial 25(OH)D level was significantly higher in males compared to females (*P* < 0.001) and as expected due to inverse relation between vitamin D and PTH, initial PTH was higher in females than males with significant difference (*P* < 0.001)|. The percent changes in 25(OH)D between initial and 4 month and between 4 month and 12 month were higher in females than males (*P* < 0.05). There was close to significant statistical difference in serum calcium percent change between initial and 4 month assays (*P* = 0.045) (Table 4).

**Table 4:** Comparison between male and female participants as regards initial{25 (OH)D, calcium, parathyroid hormone}, 4 months {25 (OH)D, calcium, parathyroid hormone, calcium/creatinie ratio},12 month 25 (OH)D, vitamin D and calcium percent changes

	Female patients (*n* = 323)	Male patients (*n* = 314)	*P* value
Initial Vitamin D(mean ± SD)	15.02 ± 7.65	17.28 ± 7.93	<0.001
Initial calcium(mean ± SD)	2.44 ± 0.12	2.45 ± 0.12	0.371
Initial PTH (mean ± SD)	51.5 ± 35.54	44.58 ± 25.45	0.005
Vitamin D after 4 Months(mean ± SD)	34.4 ± 19.74	35.1 ± 19.38	0.655
Vitamin D after 12 Months(mean ± SD)	22.14 ± 14.01	25.06 ± 14.67	0.010
Calcium after 4 Months(mean ± SD)	2.40 ± 0.11	2.39 ± 0.11	0.081
PTH after 4 M(mean ± SD)	59.72 ± 36.1	61.08 ± 34.01	0.627
Calcium/creatinine ratio (mean ± SD)	0.15 ± 0.013	0.14 ± 0.08	0.231
Initial to 4 Month vitamin D% change	187.22 ± 216.18	149.59 ± 177.09	0.017
4 Month to 12 Month vitamin D% change	˗32.62 ± 33.36	˗27.14 ± 31.65	0.034
initial to 4 Month Calcium %change	˗1.36 ± 5.75	˗2.28 ± 5.79	0.045

*P* value considered significant if < 0.05

### Comparison between participants <12 years and participants ≥12 years

Between the 2 groups with regard to all parameters except for the initial to 4 month 25(OH) percent change that was observed in subjects more than 12 years of age (*P* = 0.045) (Table 5).

**Table 5:** Comparison between participants <12 years and >12 years as regards initial{25 (OH)D, calcium, parathyroid hormone}, 4 months{25 (OH)D, calcium, parathyroid hormone, calcium/creatinine ratio},12 month 25 (OH)D, vitamin D and calcium percent changes

	participants <12 years (*n* = 509)	participants ≥ 12 years (*n* = 128)	*P* value
Initial Vitamin D(mean ± SD)	16.39 ± 7.86	15.13 ± 7.82	0.107
Initial calcium(mean ± SD)	2.45 ± 0.12	2.44 ± 0.11	0.839
Initial PTH (mean ± SD)	47.32 ± 30.47	51.17 ± 33.63	0.211
Vitamin D after 4 Months(mean ± SD)	34.26 ± 19.6	36.66 ± 19.3	0.216
Vitamin D after 12 Months(mean ± SD)	23.4 ± 14.45	24.29 ± 14.27	0.533
Calcium after 4 Months(mean ± SD)	2.39 ± 0.11	2.41 ± 0.11	0.271
PTH after 4 Months (mean ± SD)	60.59 ± 34.61	59.6 ± 36.92	0.777
Calcium/creatinine ratio (mean ± SD)	0.15 ± 0.11	0.15 ± 0.09	0.791
Initial to 4 Month vitamin D% change	160.76 ± 192.82	200.12 ± 218.12	0.045
4 Month to 12 Month vitamin D% change	˗29.74 ± 32.95	˗30.6 ± 31.41	0.788
Initial to 4 Month Calcium %change	˗1.92 ± 5.89	˗1.39 ± 5.32	0.350

*P* value considered significant if < 0.05

## Discussion

In our study, we compared 25 (OH) D in 3 groups with different modalities of vitamin D replacement over 1 year period.

Because some studies have shown rickets can manifest in patients with 25(OH)D concentration up to 20 ng/ml [22], as well as the higher values that are needed for non-bony outcomes [23], this directed us to use 30 ng/ml as a cut off value for vitamin D insufficiency. This is in agreement with studies showed that serum 25 (OH) D 30–32 ng/ml results in maximal suppression of PTH, optimal intestinal calcium absorption and prevention of fractures [24–26].

Role of diet and sun exposure as a source of vitamin D over the one year period were assumed to be insufficient. Otherwise, they did not have vitamin D insufficiency from the start and was obvious in continued drop of vitamin D in group 1.

**Fig. 1:**
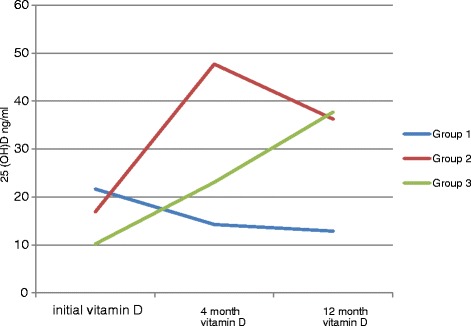
Initial, 4 month and 12 month 25 (OH) vitamin D level mean (ng/ml) in the 3groups. 25 (OH) D showed continued drop in group 1, sharp rise followed by drop in group 2 and continuous rise in group 3

Mean 25 (OH) D level progressively decreased in those who received only RDA (400 IU/day) (Fig. 1) with highest percent of deficiency after 4 months and 12 months follow up compared to other groups despite they had the highest initial 25 (OH) D level. We think it is a malpractice to treat vitamin D insufficiency with only RDA doses because it was recommended by the Institute of Medicine assuming that vitamin D is sufficient initially [27], so normalization with loading doses is initially needed. European Society of Pediatric Endocrinology (ESPE) recommends a dose of 400 IU calciferol daily for a child of any age [6]. However our results doubt the efficacy of dose 400 IU as daily replacement to maintain 25 (OH) D level as it started to drop after reaching highest values when maintained on it over 8 months in group 2 (Fig. 1). Also noted that these recommendations only provide sufficient vitamin D to prevent osteomalacia and rickets [28] and such an intake alone, in the absence of skin synthesis, will not provide optimal status. Accordingly, several learned bodies have recently increased their recommendations for vitamin D intake [29].

In group 2, the 4 month dose was 384.000 IU, which appears acceptable if compared to recent clinical approaches in adults showing regimens >600.000 IU administered over 2 months with 25 (OH) D reaching sufficient levels [30]. Also in reference to new tolerable upper intake level of 4000 IU/day [23], using 2000 IU daily then 1000 IU for 3 months then 9 months periods respectively in group 3 over 1 year period of the study is acceptable.

With 4 months follow up, mean of PTH was high in groups 1 and 3 which can be explained by the continuous drop of vitamin D in group 1. However, the high PTH level in group 3 cannot be explained based on vitamin D levels because vitamin D mean after 4 months is acceptable, but may be related to the rate of rise of vitamin D. Also, percent of sufficient participants was 36.8% in group 2 (dose 384.000 IU) while was 1.5% in group 3 (dose 210.000 IU) which is nearly half the dose in group 2. So for a mere aim to normalize 25 (OH) D, higher doses may be needed. Adult regimens of at least 600000 IU ergocalciferol appeared to be the most effective in achieving vitamin D sufficiency [6], and comparable doses are expected in pediatrics however further studies needed. In group 2, six patients developed vitamin D excess (i.e., > 100 ng/ml) [31], this can be explained by their age was less than 6 years, average daily intake was 3200 IU which exceeds the tolerable upper intake level for this age (3000 IU) [32].

In our study, we extended follow up till 12 months to evaluate which regimen was effective in maintaining acceptable vitamin D levels. However the total dose over 1 year was almost the same in group 2 (480.000 IU) and group 3 (450.000 IU), yet percent of deficient participants was 76.1% in group 2 compared to 91.2% insufficient in group 3. This is against the opinion raised by pepper et al., 2009 [30] that what is more predictive of vitamin D sufficiency is the total dose of vitamin D supplement rather than the frequency of dosing. This simply can be explained by the fact that any pharmaceutical product has a half life.

Our study showed privilege of regimen used in group 3 over the one used in group 2 as it shows a continuous steady rise of 25 (OH) D over the 1 year period (Fig. 1), no reported vitamin D toxicity in group 3, median percent change of calcium after 4 months was +1.2% in group 2 reaching border line high values, significantly higher calcium creatinine ratio in group 2 and re-rise of percent of deficient participants with 12 months follow up. However, Compliance with long term vitamin D supplementation is often poor: one off, high dose oral or intramuscular therapy is an effective option if concordance is suspect [33].

Because of the concepts of sun avoidance and religious traditions, females spend more indoor hours and cover most of the body when going outdoor, initial and 12 month 25 (OH) D assay were significantly lower in females.

Strengths of the study: It is the First study to compare the fluctuations of vitamin D level over 1 year period comparing 3 different regimens (Fig. 1).

Limitations of the study: Included children and adolescents come from hot gulf area with indoor hours during daytime more than their peers of other world regions. So our findings may not be generalizable to other regions, however vitamin D insufficiency is an ignored epidemic all over the world but more in South Asia and Middle East [34]. Our study missed assay of biochemical bone markers at 12 months follow up. It will be valuable to compare between the effect of rapid rise and the insidious rise of vitamin D on these markers.

## Conclusions

Till now, no standard regimen is available for treatment of vitamin D insufficiency. We recommend low loading dose of vitamin D around 100.000 to 200.000 IU with high maintaince dose of 1000 IU as a way of replacement rather than initial high dose and a low maintaince dose as the later can cause temporary rise of 25 (OH) D and hypercalcemic side effects.

## Abbreviations

25(OH)D: 25 hydoxy vitamin D
AAP: American Academy of Pediatrics
BMI Z score: Body mass index Z score
CDC: Centre for Disease Control
ESPE: European Society of Pediatric Endocrinology
IOM: Institute of Medicine
KDOQI: Kidney Disease outcomes Quality initiative
PTH: Parathyroid hormone
RDA: Recommended dietary allowance
Vitamin D2: Ergocalciferol
Vitamin D3: Cholecalciferol
